# Supplementary material for: Evaluation of Native Entomopathogenic Fungi for the Control of Fall Armyworm (Spodoptera frugiperda) in Thailand: A Sustainable Way for Eco-Friendly Agriculture
Source: J Fungi (Basel). 2021 Dec 13;7(12):1073. doi: 10.3390/jof7121073 (PMC8705808; doi:10.3390/jof7121073)
Supplement: Supplementary file 1 [file jof-07-01073-s001.zip › jof-1487733-supplementary.pdf]

Journal of Fungi

Supplementary Table S1. The macroscopic and microscopic colony characteristics of the six isolates of *Beauveria bassiana.*

Isolate	Colony Characteristics					Conidial	
	*Colour	Shape	Growth Pattern	Elevation	Texture	Length (μm)	Width (μm)
BCMU1	White/Brown	Round	Disperse	Raised	Smooth	2.523±0.147 abc	1.685±0.097 a
BCMU2	White/Yellowish brown	Round	Dense and disperse	Raised	Smooth	2.286±0.077 a	1.689±0.071 a
BCMU3	White/Brown	Oval	Dense Disperse	Raised	Smooth	2.749±0.126 bc	2.165±0.079 c
BCMU4	Off-white/Yellowish	Round	Disperse	Raised	Smooth	2.412±0.092 ab	1.880±0.048 ab
BCMU5	White/Brown	Oval	Disperse	Raised	Smooth	2.872±0.103 c	2.059±0.064 bc
BCMU6	White/Yellowish-brown	Round	Dense and disperse	Raised	Smooth	2.885±0.076 c	2.032±0.069 bc

*Colony color on the top and reverse side.
